# Polymorphic hyperkeratotic plaques in a patient with AIDS

**DOI:** 10.1016/j.jdcr.2026.05.055

**Published:** 2026-06-02

**Authors:** Juris Podoļanskis, Laura-Agnese Pauliņa, Anastasija Siča, Kristīne Ābeltiņa, Kristīne Nevidovska, Artūrs Kaļva, Lāsma Kalnbērza

**Affiliations:** a Department of HIV/AIDS (Dermatology-Venerology), Riga East University Hospital, Latvian Centre of Infectious Diseases, Riga, Latvia; b Riga East University Hospital, Riga, Latvia; c Riga Stradiņš University, Dermatovenerology Residency Program, Riga, Latvia; d University of Latvia, Dermatovenerology Residency Program, Riga, Latvia; e Department of HIV/AIDS, Riga East University Hospital, Latvian Centre of Infectious Diseases, Riga, Latvia; f Department of Pathology, Riga East University Hospital, Centre of Pathology, Riga, Latvia; g Institute of Public Health, Riga Stradiņš University, Riga, Latvia

**Keywords:** AIDS, dermatopathology, general dermatology, HIV, hyperkeratosis, keratoderma, scaling

## Case

**Fig 1:**
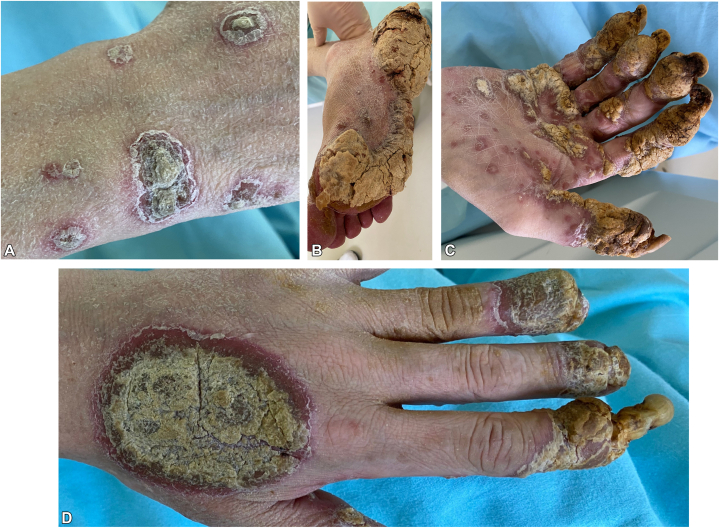
Clinical presentation of polymorphic hyperkeratotic plaques. **(A)** Hyperkeratotic plaque on the upper extremity. **(B)** Marked hyperkeratosis of the plantar surface. **(C, D)** Palmar keratoderma with nail dystrophy and a hyperkeratotic plaque on the dorsal surface of the hand.

**Fig 2:**
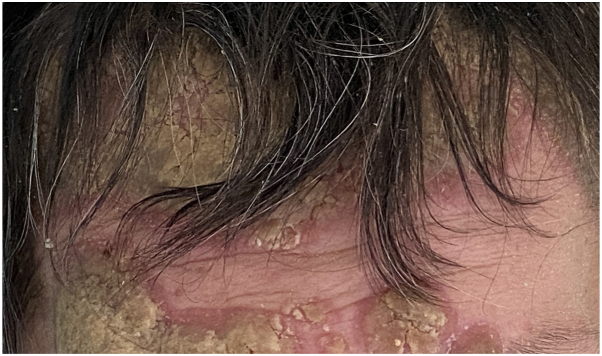
Clinical presentation of hyperkeratotic yellow plaques on the scalp and forehead. Similar lesions were also observed on the ear.

A man in his mid-40s with HIV infection, not receiving antiretroviral therapy, presented with severe palmoplantar keratoderma, nail dystrophy, dactylitis, and multiple well-demarcated, dull-red hyperkeratotic to conical plaques involving the extremities, scalp, ears, and face (Fig 1, A-D; Fig 2). A comprehensive clinical history could not be obtained due to the patient’s condition.

Laboratory evaluation revealed a CD4+ count of 5 cells/μL, pancytopenia, and negative syphilis serologies.

**Fig 3:**
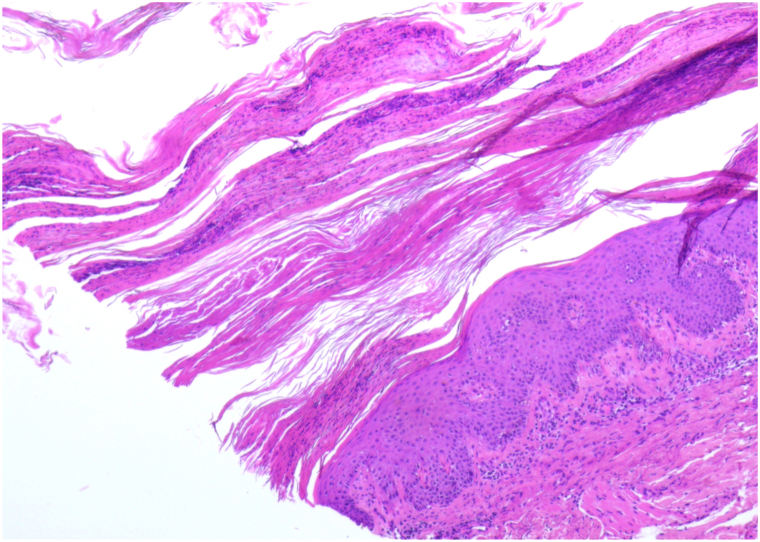
Histopathology of a skin biopsy (H&E stain, 10 × magnification). The specimen shows marked hyperkeratosis with areas of parakeratosis containing neutrophilic aggregates. The epidermis demonstrates acanthosis with focal loss of the granular layer and subcorneal neutrophilic collections. The papillary dermis showed dilated blood vessels with a perivascular lymphocytic infiltrate.

Skin scrapings from the scalp and face demonstrated an increased density of *Demodex* mites (44 mites/cm^2^; normal range <5 mites/cm^2^). Two skin biopsies obtained from the lower extremities similarly demonstrated the histological features shown in Fig 3.

Radiographs of the hands demonstrated early tapering of the distal phalanges, soft tissue swelling involving digits II to IV of both hands, and a small erosion of a distal interphalangeal joint of the right hand. Additional imaging of the chest and abdomen revealed right lower lobe pneumonia with consolidation, liver cirrhosis, splenomegaly, portal hypertension, and ascites.

### Question: Which of the following is the most likely diagnosis?

**A.** Acrokeratosis paraneoplastica (Bazex syndrome)
**B.** Crusted scabies
**C.** Hyperkeratotic psoriasis
**D.** Keratoderma blennorrhagicum
**E.** Secondary syphilis

### Answer discussion

**The correct answer is C.** Considering the clinical, laboratory, and histopathologic findings, this patient’s presentation was consistent with the hyperkeratotic variant of psoriasis in the context of HIV/AIDS. The umbrella term hyperkeratotic psoriasis encompasses elephantine-, rupioid-, and ostraceous-like morphologies.^1^ Rupioid psoriasis presents with limpet-like, cone-shaped hyperkeratotic plaques, ostraceous psoriasis with thick oyster shell-like scales, and elephantine psoriasis with large hyperkeratotic plaques, mainly on the lower limbs.^2^^,^^3^ The rupioid-like and ostraceous-like morphologies are shown in Fig 1, A and Fig 1, B-D, respectively.

This case demonstrates that severe HIV-associated psoriasis can present with multiple co-existing hyperkeratotic morphologies and joint involvement, supporting the concept of a broad morphological continuum in immunosuppression-associated disease.^4^ HIV-associated psoriasis is often characterized by greater severity, atypical clinical presentations, treatment resistance, and a higher frequency of psoriatic arthritis.^4^

The high density of *Demodex* mites in our patient may have been related to severe immunosuppression, which is more commonly observed in patients with CD4 counts below 200 cells/mm^3^.^5^ Demodicosis may also worsen after initiation of ART due to immune reconstitution inflammatory syndrome (IRIS).^5^ In our case, ART was not initiated.

Given the severity of the patient's immunosuppression, dermatologic management was conservative. Treatment consisted of topical high-potency corticosteroids, salicylic acid, and clotrimazole for scalp and facial sebopsoriasis-like involvement. Partial improvement was noted during treatment, with plaques loosening and thinning.

Optimal management for hyperkeratotic psoriasis in patients with HIV would include initiation of ART, topicals (such as corticosteroids, keratolytics, vitamin D analogues, and retinoids), and phototherapy.^6^ Acitretin would be a preferred systemic option given the hyperkeratotic presentation and its non-immunosuppressive profile. For refractory disease, apremilast or biologic therapies (TNF-α, IL-12/23, and IL-17 inhibitors) may be considered in patients with stable HIV under close monitoring.^6^ Immunosuppressive agents such as cyclosporine or methotrexate may be reserved for cases in which previous therapies are ineffective or unavailable.^6^

The patient later died during hospitalization due to progressive multisystem complications associated with advanced HIV/AIDS.

## Conflicts of interest

None disclosed.
